# Vaginal microflora following the use of a disposable home-use vaginal device and a commercially available ring pessary for pelvic organ prolapse management: a randomized controlled trial

**DOI:** 10.1007/s00404-023-07260-w

**Published:** 2023-10-26

**Authors:** Elan Ziv, Nathan Keller, Tsvia Erlich

**Affiliations:** 1 ConTIPI Medical Ltd, 2 Alon Ha’ Tavor St, Southern Industrial Park, 3088900 Caesarea, Israel; 2 Ariel University, Ariel, Israel; 3 Sheba Medical Center, Ramat Gan, Israel

**Keywords:** Disposable vaginal device, Nonsurgical management, Pelvic organ prolapse, Pessaries, Vaginal microflora

## Abstract

**Purpose:**

To investigate whether ProVate, a novel, disposable, self-inserted vaginal device for pelvic organ prolapse management, clinically affects the vaginal microflora, as compared with a commercially available ring pessary, to assess its microbiological safety.

**Methods:**

This interventional, prospective, multi-center, open-label, randomized, controlled, statistically powered (noninferiority), home-use, crossover study was conducted at seven sites. Participants were randomized into either group A (using ProVate and then a new reusable commercially available ring pessary [control]) or B (using control device and then ProVate) with a 1:1 ratio. Noninferiority of ProVate over the control was evaluated for the primary endpoint, which was based on meeting one of the failure criteria: significant change in *Lactobacillus* spp*.*, *Gardnerella vaginalis*, *Candida* morphotypes, or *Staphylococcus aureus* levels compared to the baseline (significant change: Nugent score ≥ 7 or > 1 scale unit increase in *S. aureus* or *Candida* morphotype), bothersome vaginal infection symptoms, or symptoms requiring treatment for infection.

**Results:**

The study included 58 participants (mean age: 64.5 years, 91.4% postmenopausal). There were no significant microfloral changes in terms of the four microorganisms mentioned above, the rate of Nugent score ≥ 7 after use was low and comparable between the two devices, and the rate of patients with a > 1 unit-scale change (increase or decrease) from the baseline to the end-of-use phase in any studied microorganism was comparable between the devices. The failure rate was 15.5% for ProVate and 15.5% for control while using 383 ProVate devices over 1647 days or one control device throughout the study. Two patients had bothersome vaginal complaints and one had overt vaginal infection in the control group, but no such cases were observed in the ProVate group.

**Conclusion:**

The primary endpoint of possible vaginal microbial changes, bothersome vaginal symptoms, or treatment-requiring vaginal complaints while using ProVate was successfully met. Our findings show that the vaginal microflora is comparable when using either ProVate or commercially available ring pessary (control) with a relatively low rate of vaginal infections.

**Trial registration details**: ClinicalTrials.gov; URL: https://www.clinicaltrials.gov/ct2/show/NCT03345121?term=NCT03345121&draw=2&rank=1; No. NCT03345121; Registration date, November 17, 2017; initial enrollment started on August 20, 2017.

**Supplementary Information:**

The online version contains supplementary material available at 10.1007/s00404-023-07260-w.

## What does this study add to the clinical work?

**Table:** 

The study provides new insight into the variations of vaginal microflora while using pessaries in general. It demonstrates that the new, disposable, single-use ProVate ring pessary and a new, unused commercially available reusable pessary do not have any clinically meaningful impact on the vaginal microflora and are safe for use.	

## Introduction

The reported prevalence of pelvic organ prolapse (POP) is very high, widely varying depending on whether it is reported by symptoms (1%–31%), pelvic examination (10%–50%), or both (20%–65%) [1]. However, only a few women eventually approach their physicians for symptomatic POP [2], mainly due to reluctance to disclose their problem. Most women require only nonsurgical POP management, and many international guidelines consider pessaries as the primary treatment. Vaginal pessaries are used for management of most POP stages, and the currently available ring pessary (control) is widely used [3, 4]. Currently, pessary use is associated with various adverse events [5], cumbersome insertion and removal, occasional pain, adverse influence on sexual activity, and requiring periodic involvement of a medical practitioner for many years. Therefore, many POP patients refrain from using them and look for alternatives (e.g., surgery), or may not undergo treatment at all.

**Fig. 1:**
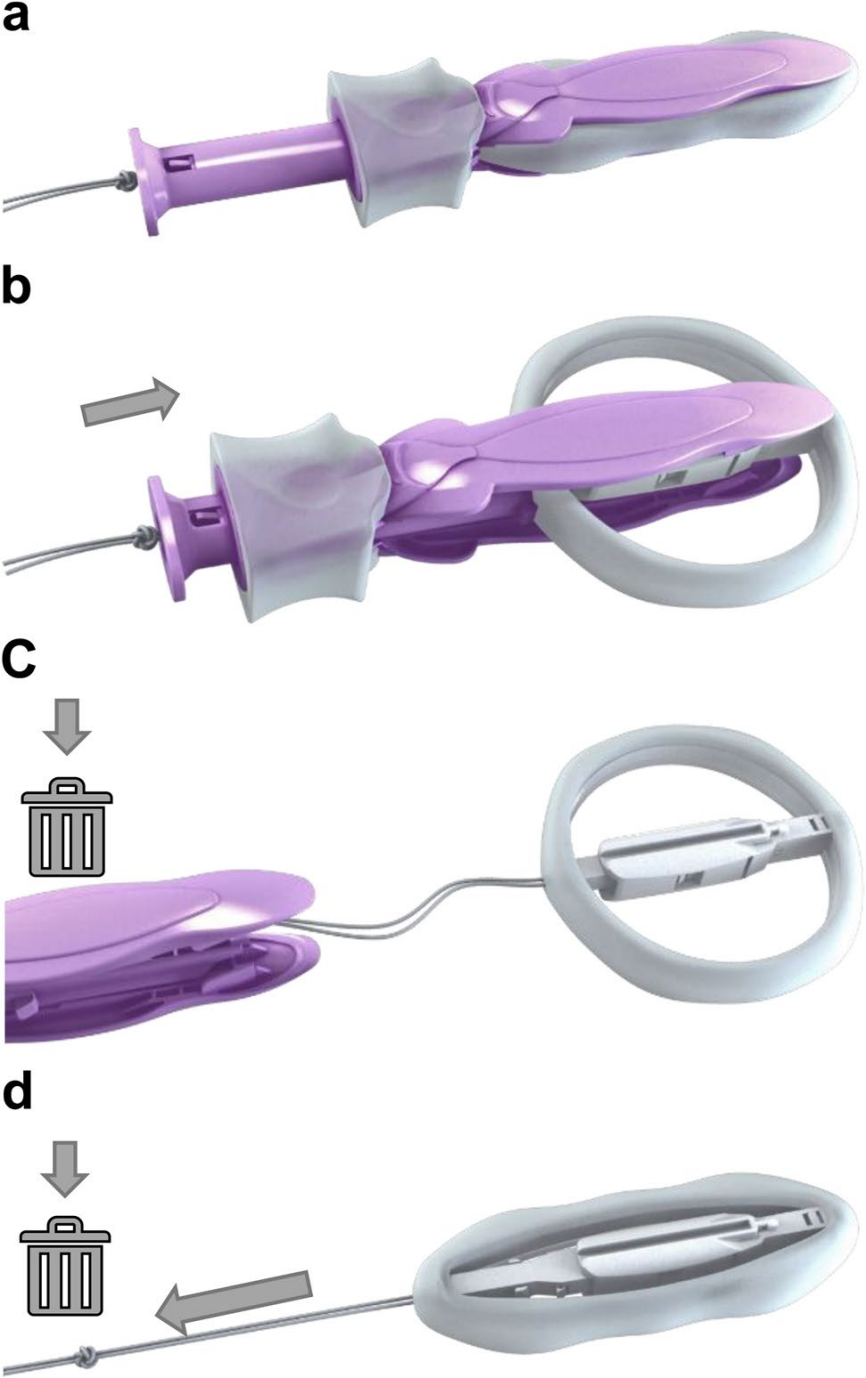
The ProVate device with its various configurations during insertion and removal. The ProVate device is provided clean, within a personal wrap and with a disposable applicator, readily available for immediate vaginal insertion in small dimensions (**a**). Following vaginal insertion, similar in concept to a menstrual tampon insertion, the plunger is pushed and the slender compacted device within the applicator gradually enlarges to become a ring (**b**). After pushing the plunger, the ring becomes fully deployed and the applicator separates from the ring, and is removed from the vagina for disposal, leaving the string available for later removal (**c**). The deployed ring may remain in the vagina for up to 7 days, when a pull on the string collapses the ring into its slender pre-insertion size, allowing for comfortable removal and disposal (**d**)

The ProVate device (Fig. 1), covered by soft biocompatible elastomer, was designed to function similarly to a ring pessary and provide adequate support to prolapsing organs, while overcoming most of the disadvantages of existing pessaries [6] as it is disposable, intended for home use, and inserted vaginally in small dimensions, whenever and wherever a woman desires, within a disposable applicator. Following insertion, the device opens into a ring while the applicator is disposed of ProVate can be used for up to 7 days, and when the removal string is pulled, the device returns to its slender pre-insertion shape for easy removal and disposal, which can increase its use rate among women for POP management.

The vaginal microflora in healthy premenopausal women is dominated by lactobacilli [7], which protect the host from urogenital infections. Vaginal microbiomes are unstable and subject to many influences that alter both their character and composition and may cause daily fluctuations. These influences include hormonal changes, stress, douching [8], menses [9], spermicides, medications [10], and sexual intercourse [11, 12]. Menopause induces further changes in the vaginal microbiome composition, mainly the predominance of *Gardnerella vaginalis*, *Ureaplasma urealyticum*, *Candida albicans*, and *Prevotella* spp*.*, along with a decrease in *Lactobacillus* spp. [13]. These alterations may occur often [14] and are mostly transient and are not indicative of, or necessarily followed by, symptomatic vaginal infection [15].

Whether vaginal devices cause meaningful changes in the microflora and increase the infection rate has been the subject of much research [16, 17]. For many years, pessaries were considered a major cause of vaginal discharge and infections [18] when used. However, for the most widely used devices (e.g., ring POP pessaries and contraceptive rings), there is no compelling evidence that the vaginal microflora is considerably affected [19, 20].

The present randomized controlled trial tested whether ProVate device, being a single-use device used for a short term only, meaningfully alters the vaginal microflora, compared with a commercially available ring pessary. Specifically, changes in *Lactobacillus* spp*.*, *G. vaginalis*, *Candida* morphotypes, and *Staphylococcus aureus* levels from baseline levels, alterations in the Nugent score, and bothersome or treatment-requiring vaginal symptoms were assessed before and during the use of ProVate and control devices.

## Materials and methods

### Study design

This interventional, prospective, multicenter, open-label, randomized, controlled, home-use trial tested ProVate and control devices in a sequential crossover fashion.

**Fig. 2:**
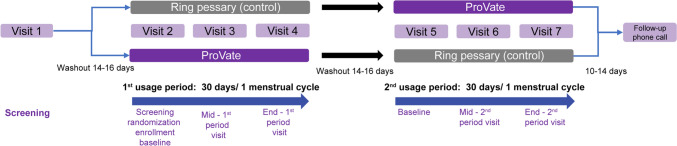
Study design. Screening started at visit 1, followed by a 14–16-day washout period. Visit 2 was a baseline visit for the 1st use period, with screening, randomization into either the ProVate or control group, and enrollment of eligible participants. Visit 3 was a mid-period visit to assess for compliance with study restrictions. Visit 4 was the end-visit of the 1st use period, and results obtained from this visit were compared with those obtained from visit 2. Following another 14–16 days of the washout period, the 2nd use period began, where each woman used the alternative studied device (visits 5–7), for the same length of time. A follow-up phone call to the women at 10–14 days after visit 7 marked the end of the study

Following screening (Fig. 2), the first use period began after a 14–16-day washout period, in which participants refrained from using any vaginal device and complied with study restrictions. During visit 2, screening was completed by verifying participants’ ability to retain one of the available sizes of ProVate or control device. Participants were recruited from seven gynecology/urogynecology community clinics and were randomized into group A (using ProVate and then a new reusable commercially available ring pessary [control]) or B (using a control device and then ProVate) in a 1:1 ratio. Participants received either a clean-sealed disposable ProVate or a new reusable ring pessary made by a single US manufacturer (Ring with support by Milex, Cooper Surgical Inc., Trumbull, CT). Visit 3 was an interim visit to assess correct sizing and adherence to study restrictions. Following another same-length washout period after visit 4, the 2nd use period commenced, having the same chain of events but with an alternate device. Size fitting was performed at the beginning of each use period, lasting for 30 days (± 3 days) for postmenopausal participants, or the length of participants’ menstrual cycle (± 3 days, range: 26–40 days) for menstruating participants. Participants were allowed to use as many ProVate devices as they wished, for at least 24 h and up to 7 days each.

Symptoms were assessed during vaginal examinations and study visits, as well as based on the use diary filled out by the user.

Vaginal microflora test samples were collected from all eligible participants at the beginning and end of each use period. All samples were analyzed at a central laboratory, Covance Labs (currently LabCorp).

### Inclusion/exclusion criteria and study restrictions

This study was conducted between August 2017 and September 2018. Participants were included if they were women, aged 21–80 years, diagnosed with POP stages 2–4, according to the POP quantification (POP-Q) [21] in one or more sites along the vaginal walls, had used any ring pessary, able to use both hands and insert a vaginal device, able to retain a 61–91 mm pessary, and willing to comply with study restrictions.

Women were excluded if they had been previously unable to tolerate use of tampons or vaginal pessaries, were currently enrolled in another clinical study, had morbid conditions or severe systemic disease that could limit their participation, were pregnant or suspected of being pregnant or intended to be pregnant during the study course, had abnormal vaginal bleeding in the past 6 months, vaginal surgery during the preceding 3 months, severe vaginal atrophy, existing vaginal or vulvar laceration, symptomatic vaginal or urinary tract infection as determined by physical and laboratory tests, recurrent urinary tract infections, and abnormal cervical cytology, were currently using corticosteroids or antibiotics, or had current medical conditions that may have compromised their immune system.

Study restrictions: all participants were instructed to avoid activities or commercial products that may impact the vaginal microflora, including vulvar or intimate cosmetics, medications, contraceptives, wipes, and any vaginal devices other than the study devices. They were also instructed to use only the menstrual supplies and condoms provided at the study site and refrain from intercourse for 48 h before microflora sampling.

### Endpoints

The primary endpoint was one of the failure criteria: a significant change in four key microorganisms, *Lactobacillus* spp*.*, *G. vaginalis*, *Candida* morphotypes, or *S. aureus*, where a significant change (“failure criterion”) was defined as either (I) Nugent score ≥ 7, or > 1 scale unit increase in *S. aureus* or *Candida* morphotype; (II) bothersome vaginal symptoms; or (III) vaginal symptoms requiring treatment.

The secondary endpoints were as follows: (I) the proportion of participants with ≥ 1-unit-scale changes in microbial counts following device use as compared to baseline in any of the four study microorganisms; (II) proportion of participants with a Nugent score ≥ 7 following device uses; and (III) proportion of participants with vaginal infection symptoms that were bothersome or required treatment for infection.

### Microflora analysis

Evaluation was performed using Gram stain counts of colony forming units/high power field (CFU/HPF) for *Lactobacillus* spp*., G. vaginalis* and *Candida* morphotypes, and semi-quantitative blood agar culture plate counts for *S. aureus.* The *Lactobacillus* spp*.* score range was 0–4 [0 =  > 30 CFU/HPF and 4 = no colonies], whereas other microorganisms were scored in reverse [0 = no colonies at all and 4 =  > 30 CFU/HPF].

The changes in Gram stain semi-quantitative counts for each of the four microorganisms before ProVate or control device insertion (visits 2/5) as compared to the end of the use period (visits 5/7) were recorded as either beneficial, non-beneficial, or no change, using a 0–4 scale. No change was defined as a score of + 1 to–1, and a meaningful change as a change in score of + 2– + 4 or − 2– − 4 (beneficial or non-beneficial for each microorganism).

### Statistical analyses

Sample size calculations were based on the primary endpoint, and estimations were based on a previous study [22]. Approximately 14.6% of participants would have a significant change in *G. vaginalis* and *Lactobacillus* spp. levels*,* and 2.4% of participants would have a significant increase in *Candida* spp. levels. Additionally, a 3% increase in *S. aureus* growth was estimated and up to 17% of participants would experience bothersome vaginal symptoms (unpublished data). Conservatively, participants would experience an overall 20% change in microflora, and 2%–8% would have an infection with microorganisms of interest that would require treatment. Assuming a failure rate in the control group of 30% and an actual difference in failure rate between groups of zero, a sample size of 54 participants would achieve 80% power at a significance level of 0.025 using a one-sided non-inferiority test of correlated proportions (McNemar test assuming 10% discordant pairs), and a non-inferiority margin of 15%.

The full analysis (FA) set included all eligible participants who used at least one device (even if the insertion process was never completed) and served as the principal analysis set for the safety assessment. The per-protocol (PP) analysis set included all participants from the FA set who had used the study devices for at least 16 days without considerable protocol deviation and served as the principal analysis set for the primary and secondary endpoint analyses. Two-tailed tests were performed for all analyses with a significance level of 0.05. Differences were considered statistically significant at *P* ≤ 0.05.

As the primary endpoint, the proportion of participants who met the failure criteria was evaluated for non-inferiority of ProVate vs. the commercially available vaginal ring pessary (control). Non-inferiority of ProVate is present if the upper limit of a one-sided 97.5% confidence interval constructed on the difference in proportions (ProVate—commercial vaginal ring pessary), considering the correlation due to the crossover design, is less than the noninferiority limit of 15%.

For the secondary endpoints, the proportion of participants treated with each device meeting each individual failure criterion and the proportion of participants with an increase in microbial count > 1 unit-scale in any study organism are described.

## Results

**Fig. 3:**
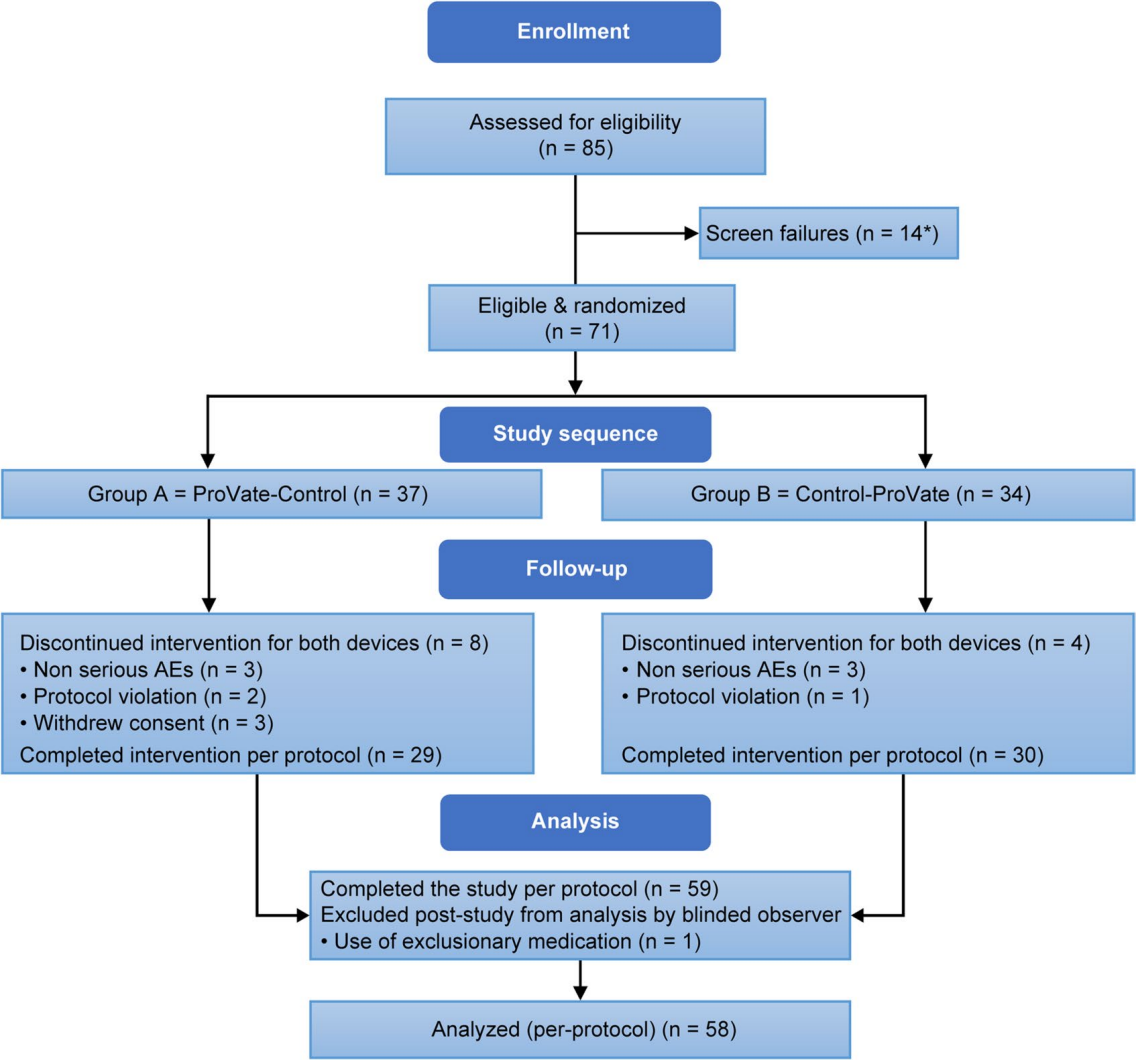
Disposition of women during the study. Altogether, 85 women were enrolled and screened, 73 were randomized into either group A (ProVate–Control) or B (Control-ProVate), and 59 participants completed the study per protocol. However, data of one woman was excluded by a blinded reviewer following the study as she used an exclusionary medication. Hence, only the data of 58 participants was analyzed per protocol. *Two participants were randomized but did not meet the inclusion criteria requiring ability to use one of the available sizes in the study (ProVate or control); hence, they were considered screen failures

Symptomatic participants accustomed to using ring pessaries were recruited from seven outpatient gynecology/urogynecology clinics (six in the US and one in Israel); altogether, 85 women with POP were screened (Fig. 3), 71 were randomized, and 58 completed the study PP analysis. Table 1 shows the participants’ characteristics.

**Table 1:** Characteristics of the study participants

		Participant’s overall characteristics [Group A/Group B]**
Mean age (Years)		64.5 ± 10.57 [64.6 ± 10.9/64.5 ± 10.46]
Body mass index		28.2 ± 5.5 [29 ± 6.36/27.6 ± 4.64]
Menopausal status (*N*,%)	Postmenopausal	53 (91.4%) [24 (41%)/29 (50%)]
	Premenopausal	4 (6.9%) [1 (1.72%)/3 (5%)]
	Perimenopausal	1 (1.7%) [1 (1.7%)/0 (0%)]
Systemic HRT* use (*N*,%)		4 (6.9%) [3 (5%)/1 (1.7%)]
Vaginal estrogen use (*N*,%)		5 (8.6%) [1 (1.7%)/4 (6.9%)]
POPQ-Q staging	2	21 (36.2%) [12 (20.7%)/9 (15.5%)]
	3	35 (60.3%) [13 (22.4%)/22 (38%)]
	4	2 (3.4%) [1 (1.7%)/1 (1.7%)]

^*^HRT Hormone Replacement Therapy. **Group A used ProVate and then the control, and Group B used the control and then ProVate

Altogether, 383 ProVate devices were used (average: 5.7 ± 1.5 devices/user) in the FA set, whereas, in the PP set, 350 ProVate devices were used (average: 6.0 ± 1.1 devices/user). The average device use length per subject in the PP population was 28.4 ± 3.58 days for the ProVate device and 29.9 ± 2.66 days for the control device. In the study, most use events (62.9%) of a single ProVate device lasted for at least 4 days, and 36.8% of use events lasted for 6–7 days. A single reusable control device remained in the vagina during the whole control device phase (in most cases, it remained in the vagina for at least 22 days).

The total number of use days was 1647 and 1734 for ProVate and control device, respectively.

No significant effect of randomization sequence was observed (*P* = 0.325), suggesting a lack of sequence effect and allowing pooling of the results for each device from both sequences in all analyses.

### Primary endpoint

The total number of participants who met at least one failure criterion was comparable between ProVate and control groups. The failure rate was 15.5% for both ProVate and control devices [95% confidence interval − 13%:13%, *P* > 0.999], which was lower than the estimated rate of 30%, with a one-sided 97.5% upper limit of 13% (within the non-inferiority limit of 15%).

### Secondary endpoints

There was a high rate of fluctuations in the vaginal microflora, ranging from 36.2% to 44.8% (Table 2). Although objective laboratory results showed no meaningful difference between the results of ProVate and control devices, the control group had slightly more bothersome vaginal complaints (two women, 3.4%) and one case requiring treatment (one woman, 1.7%), whereas there were no such cases in the ProVate group (Table 2).

**Table 2:** Secondary endpoints—microflora changes and reported symptoms

Failure criteria	Usage period	
	ProVate (*n*,%)	Control (*n*,%)
Patients with a > 1 unit-scale change (increase or decrease) from baseline to end-of-use phase in any studied microorganism	26 (44.8%)	21 (36.2%)
Patients with Nugent score ≥ 7 following device use	4 (6.9%)	2 (3.4%)
Patients with a > 1 scale unit increase following device use in *Staphylococcus aureus*	2 (3.4%)	3 (5.2%)
Patients with a > 1 scale unit increase following device use in *Candida* morphotype	3 (5.2%)	2 (3.4%)
Patients with vaginal symptoms that are bothersome	0 (0.0%)	2 (3.4%)
Patients with vaginal symptoms related to vaginal infection that require treatment	0 (0.0%)	1 (1.7%)
Patients with a conclusion of vaginal infection (i.e., physician reported an overall conclusion of vaginal infection)	0 (0.0%)	2 (3.4%)

### Microorganism count fluctuations

Although fluctuations in microorganism counts for both devices were as high as 44%, they were almost balanced, and in most cases, only a slight score change was observed (Table 3). The same balance was observed with the Nugent score, where changes above and below the threshold of infection (≥ 7) were similar.

**Table 3:** Possible microfloral changes while using ProVate and control device

Microorganism	Change	*n* (%), *N* = 58, per protocol	
		ProVate	Control
*Lactobacillus* spp.	Beneficial (+ 2, + 3, + 4)	5(8.6%)	12(20.7%)
	No change (− 1,0, + 1)	47(81.0%)	45(77.6%)
	Non-beneficial (− 2, − 3, − 4)	6(10.3%)	1(1.7%)
*Gardnerella vaginalis*	Beneficial (− 2, − 3, − 4)	6(10.3%)	5(8.6%)
	No change (− 1,0, + 1)	41(70.7%)	48(82.8%)
	Non-beneficial (+ 2, + 3, + 4)	11(19.0%)	5(8.6%)
*Staphylococcus aureus*	Beneficial (− 2, − 3, − 4)	0 (0%)	0 (0%)
	No change (− 1,0, + 1)	56(96.6%)	55(94.8%)
	Non-beneficial (+ 2, + 3, + 4)	2(3.4%)	3(5.2%)
*Candida* morphotypes	Beneficial (− 2, − 3, − 4)	0 (0%)	0 (0%)
	No change (− 1,0, + 1)	55(94.8%)	56(96.6%)
	Non-beneficial (+ 2, + 3, + 4)	3(5.2%)	2(3.4%)
Nugent Score	No change	53(91.4%)	52(89.7%)
	From normal (< 7) to abnormal (≥ 7)	4(6.9%)	2(3.4%)
	From abnormal (≥ 7) to normal (< 7)	1(1.7%)	4(6.9%)

No significant microflora change, beneficial or non-beneficial, was recorded between the new disposable ProVate device and a fresh reusable commercially available ring pessary. No patient using ProVate had vaginal infections or bothersome symptoms, or required treatment (383 devices with 1647 use days, maximum 7-day use), whereas in the control group, one patient had overt vaginal infection, two had signs of infection or bothersome symptoms (requiring antibiotic treatment), and two had urinary tract infections.

## Discussion

This study was intended to confirm that ProVate does not cause meaningful changes in vaginal microflora compared to the control and that microfloral fluctuations were comparable and can be attributed to regular vaginal microflora alterations unrelated to the use of vaginal devices. The primary and the secondary endpoints were successfully met, indicating that ProVate does not alter the vaginal microflora in a clinically significant manner compared with a ring pessary (control). These results are consistent with those of previous studies [23, 24], which examined vaginal microflora and complaints while using pessaries, and concluded that there were more complaints while using pessaries (e.g., discharge) but no evidence of microflora changes.

Common practices with existing ring pessaries include their 3–12-month use, removal, cleaning, and reinsertion. Previous data support that vaginal devices do not promote vaginal infections and that vaginal microflora is unstable, with marked variability in the identity and abundance of various microorganisms, irrespective of the presence of a device. Increased vaginal infections were found while using vaginal devices, affecting 6%–33% of users [25]. This inconsistency in the literature may be attributed to the definition of vaginal infection and determinant factors used (e.g., changes in microflora, vaginal complaints, discharge, thrush, pain, and so on). Therefore, adherence to the guidelines from CDC (https://www.cdc.gov/std/tg2015/bv.htm) and other organizations (http://www.iusti.org/regions/europe/pdf/2011/Euro_Guidelines_Vaginal_Discharge_2011.Intl_Jrev.pdf) claiming that microfloral fluctuations are not infections and do not require treatment unless accompanied by complaints and/or symptoms is required. These constant spontaneous fluctuations (reaching 44% in this study) limit the value of laboratory results in diagnosing vaginal infections; hence, clinical signs and bothersome symptoms should be the main determinants of diagnosis.

The Nugent semiquantitative scale, originally developed for premenopausal women, is a simple and accessible method for diagnosing bacterial vaginosis, allowing for easy comparison of pre- and post-results and assessment of the balance between beneficial and non-beneficial microorganisms; hence, it is applicable for any age. Although advanced methods for detecting minimal amounts of nonhuman DNA exist, simple, well-established, semiquantitative Gram stain and plate count methods were utilized to avoid test oversensitivity, which may impact the study outcome.

This study is the first to compare possible vaginal microflora changes when using a clean disposable device at every insertion (ProVate) and when a reusable device is used for the first of many usages (control), and sheds light on the following two factors not sufficiently addressed in the regular pessary use practice:

(a) the reusability of existing pessaries, and the subsequent level of cleanliness upon reinsertion is less than optimal; hence, insertion of a reusable device with possible remaining biofilms [26] may be expected; and (b) although some studies have demonstrated the ability to leave a pessary in place for over a year, others [27], who also examined microflora changes, showed that frequent pessary replacement (≥ 1 replacement/week) was associated with *Lactobacillus* predominance, a vaginal defense mechanism against infections.

In this study, the influence of device cleanliness (using only a fresh device: either disposable ProVate, or a new unused package of a control device) and use length (up to 7 and 36 days for ProVate and control devices, respectively) in promoting vaginal microflora changes and in the diagnosis of vaginal infection was examined. Although the results obtained using the two devices are comparable, it should be stressed that the control device was a new clean pessary (not used reusable pessary). This may have positively shifted the results, demonstrating fewer infections or vaginal complaints with the control device than would usually be found while using the same reusable pessary over many repeated insertions for long periods, which might be reason for low rate of vaginal infections and complaints in the control group.

The other benefits of using ProVate include the ability to perform home self-insertion using an applicator, no clinic dependency, and shift of control over the medical problem into the hands of women. Additionally, ProVate is disposable and has small dimensions during insertion and removal, short use period, and low expected rate of vaginal infections and complaints, which may contribute considerably to treatment compliance and better acceptance.

The study strengths were its randomized, controlled, multicenter design, strict supervision (including a daily diary of adverse events and device follow-up), combined objective laboratory results with subjective complaints as endpoints, and use of a central laboratory for all sites. The benefit of ProVate, without any vaginal infections or complaints when participants replaced 6–9 devices during the study period, has been well demonstrated.

One limitation of this trial is the use of control devices only during its primary round (using a new clean package), which prevented data collection regarding vaginal infections and complaints over long-term use, when biofilms accumulate and insufficient cleaning is expected. Another limitation was that only a few microorganisms (although they are responsible for the vast majority of genital infections) were examined and that study restrictions (e.g., avoidance of vulvar lotions, antibiotics, steroids, and intercourse before sampling), which differ greatly from typical behaviors, were applied.

In conclusion, the study results confirm that, compared with the control device, ProVate does not cause meaningful changes in the vaginal microflora and that microfloral fluctuations can be attributed to regular vaginal microflora alterations unrelated to vaginal device use. Together with its other features, ProVate may increase the acceptance and compliance rates among women with POP receiving nonsurgical management. Further studies without restrictions are needed to validate the results of the use ProVate and reusable ring pessaries, regardless of cleanliness and length of use.

### Supplementary Information

Below is the link to the electronic supplementary material.

Supplementary file1 (PDF 159 KB)

## Data Availability

The data that support the findings of this study are available from [ConTIPI Medical Ltd] but restrictions apply to the availability of these data, which were used under license for the current study, and so are not publicly available. Data are however available from the authors upon reasonable request and with permission of [ConTIPI Medical Ltd].

## References

[1] Brown HW, Hegde A, Huebner M, Neels H, Barnes HC, Marquini GV, Mukhtarova N, Mbwele B, Tailor V, Kocjancic E, Trowbridge E, Hayward L (2022). International urogynecology consultation chapter 1 committee 2: epidemiology of pelvic organ prolapse: prevalence, incidence, natural history, and service needs. Int Urogynecol J.

[2] Wu JM, Hundley AF, Fulton RG, Myers ER (2009). Forecasting the prevalence of pelvic floor disorders in U.S. Women: 2010 to 2050. Obstet Gynecol.

[3] McIntosh L (2005). The role of the nurse in the use of vaginal pessaries to treat pelvic organ prolapse and/or urinary incontinence: a literature review. Urol Nurs.

[4] Patel M, Mellen C, O’Sullivan DM, LaSala CA (2010). Impact of pessary use on prolapse symptoms, quality of life, and body image. Am J Obstet Gynecol.

[5] Yimphong T, Temtanakitpaisan T, Buppasiri P, Chongsomchai C, Kanchaiyaphum S (2018). Discontinuation rate and adverse events after 1 year of vaginal pessary use in women with pelvic organ prolapse. Int Urogynecol J.

[6] Ziv E, Erlich T (2022). Novel, disposable, self-inserted, vaginal device for the non-surgical management of pelvic organ prolapse: efficacy, safety, and quality of life. BMC Womens Health.

[7] Vasquez A, Jakobsson T, Ahrne S, Forsum U, Molin G (2002). Vaginal Lactobacillus flora of healthy Swedish women. J Clin Microbiol.

[8] Brotman RM, Klebanoff MA, Nansel TR, Andrews WW, Schwebke JR, Zhang J, Yu KF, Zenilman JM, Scharfstein DO (2008). A longitudinal study of vaginal douching and bacterial vaginosis–a marginal structural modeling analysis. Am J Epidemiol.

[9] Eschenbach DA, Thwin SS, Patton DL, Hooton TM, Stapleton AE, Agnew K, Winter C, Meier A, Stamm WE (2000). Influence of the normal menstrual cycle on vaginal tissue, discharge, and microflora. Clin Infect Dis.

[10] Bradshaw CS, Vodstrcil LA, Hocking JS, Law M, Pirotta M, Garland SM, De Guingand D, Morton AN, Fairley CK (2013). Recurrence of bacterial vaginosis is significantly associated with posttreatment sexual activities and hormonal contraceptive use. Clin Infect Dis.

[11] Brotman RM, Ravel J, Cone RA, Zenilman JM (2010). Rapid fluctuation of the vaginal microbiota measured by Gram stain analysis. Sex Transm Infect.

[12] Bradshaw CS, Walker SM, Vodstrcil LA, Bilardi JE, Law M, Hocking JS, Fethers KA, Fehler G, Petersen S, Tabrizi SN, Chen MY (2014). The influence of behaviors and relationships on the vaginal microbiota of women and their female partners: the WOW health study. J Infect Dis.

[13] Auriemma RS, Scairati R, Del Vecchio G, Liccardi A, Verde N, Pirchio R, Pivonello R, Ercolini D, Colao A (2021). The vaginal microbiome: a long urogenital colonization throughout woman life. Front Cell Infect Microbiol.

[14] Ravel J, Gajer P, Abdo Z, Schneider GM, Koenig SS, McCulle SL, Karlebach S, Gorle R, Russell J, Tacket CO, Brotman RM, Davis CC, Ault K, Peralta L, Forney LJ (2011). Vaginal microbiome of reproductive- age women. Proc Nat Acad Sci USA.

[15] Gajer P, Brotman RM, Bai G, Sakamoto J, Schütte UM, Zhong X, Koenig SS, Fu L, Ma Z, Zhou X, Abdo Z, Forney LJ, Ravel J (2012). Temporal dynamics of the human vaginal microbiota. Sci Transl Med.

[16] Malcolm RK, Boyd PJ, McCoy CF, Murphy DJ (2016). Microbicide vaginal rings: technological challenges and clinical development. Adv Drug Deliv Rev.

[17] Nugent RP, Krohn MA, Hillier SL (1991). Reliability of diagnosing bacterial vaginosis is improved by a standardized method of Gram stain interpretation. J Clin Microbiol.

[18] Alnaif B, Drutz HP (2000). Bacterial vaginosis increases in pessary users. Int Urogynecol J Pelvic Floor Dysfunct.

[19] De Seta F, Restaino S, De Santo D, Stabile G, Banco R, Busetti M, Barbati G, Guaschino S (2012). Effects of hormonal contraception on vaginal flora. Contraception.

[20] Ziv E, Stanton SL, Abarbanel J (2008). Efficacy and safety of a novel disposable intravaginal device for treating stress urinary incontinence. Am J Obstet Gynecol.

[21] Persu C, Chapple CR, Cauni V, Gutue S, Geavlete P (2011). Pelvic organ prolapse quantification system (POP-Q)—a new era in pelvic prolapse staging. J Med Life.

[22] Ziv E, Erlich T, Keller N (2015). A multicenter, prospective, randomized, controlled, cross-over study to assess safety, vaginal microflora changes and rate of vaginal infections while using a novel non-absorbing intravaginal device for stress urinary incontinence. Female Pelvic Med Reconstr Surg.

[23] Collins S, Beigi R, Mellen C, O'Sullivan D, Tulikangas P (2015). The effect of pessaries on the vaginal microenvironment. Am J Obstet Gynecol.

[24] Yoshimura K, Morotomi N, Fukuda K, Kubo T, Taniguchi H (2020). Changes of intravaginal microbiota and inflammation after self-replacement ring pessary therapy compared to continuous ring pessary usage for pelvic organ prolapse. J Obstet Gynaecol Res.

[25] Abdulaziz M, Stothers L, Lazare D, Macnab A (2015). An integrative review and severity classification of complications related to pessary use in the treatment of female pelvic organ prolapse. Can Urol Assoc J.

[26] Gould FG, Carey MP, Plummer EL, Murray GL, Danielewski JA, Tabrizi SN, Garland SM (2022). Bacterial biofilm formation on vaginal ring pessaries used for pelvic organ prolapse. Int Urogynecol J.

[27] Fregosi NJ, Hobson DTG, Kinman CL, Gaskins JT, Stewart JR, Meriwether KV (2018). Changes in the vaginal microenvironment as related to frequency of pessary removal. Female Pelvic Med Reconstr Surg.

